# Effects of core stability training on older women with low back pain: a randomized controlled trial

**DOI:** 10.1186/s11556-022-00289-x

**Published:** 2022-04-15

**Authors:** Le Ge, Huanjie Huang, Qiuhua Yu, Yan Li, Xin Li, Zhicheng Li, Xi Chen, Le Li, Chuhuai Wang

**Affiliations:** 1 Department of Rehabilitation Medicine, The First Affiliated Hospital, Sun Yat-sen University, Guangzhou, 510080 Guangdong Province China; 2 Institute of Medical Research, Northwestern Polytechnical University, Xian, 710072 Shanxi Province China

**Keywords:** Low back pain, Core stability exercise, Older women, Exercise

## Abstract

**Background:**

Studies have demonstrated that elderly people with low back pain (LBP) may have poor postural control compared to healthy older adults. Poor postural control is associated with poor balance performance and a high risk of serious falls. A variety of training strategies are proposed for LBP therapy, particularly core stabilization training. But this treatment for older people with LBP remains unclear.

**Methods:**

31 participants were randomly placed in a core stability training group (TG, *n* = 15) and a control group (CG, *n* = 16). The participants in the training group were required to complete 4 sets of core stability training and conventional physiotherapy 4 times per week for 4 weeks, whereas the participants in the control group only completed physiotherapy 4 times per week for 4 weeks. Ultrasound imaging was used to measure transverse abdominal muscle (TrA) thickness before and after the intervention. A 10-cm visual analog scale (VAS), the Oswestry Disability Index (ODI), and mobility functions were applied before and after the intervention. Data are reported as the median and range and were compared using two-way repeated-measures ANOVA,t-tests and chi-squared tests. *P* < 0.05 was considered significant in all statistical tests.

**Results:**

After intervention, there was a statistically significant difference in scores in the intervention group, especially for VAS, ODI, timed up-and-go,10-m walking and the four-square step test. TrA thickness was increased after core stability training, which was not observed in the control group.

**Conclusion:**

Core stability training is an effective intervention for older women with LBP.

**Supplementary Information:**

The online version contains supplementary material available at 10.1186/s11556-022-00289-x.

## Introduction

Low back pain (LBP) is one of the major disabling health conditions among older adults aged 60 years or older [1]. A study found that 62.5% of older people living in institutions suffer from LBP [2]. People with LBP frequently experience impairment of dynamic balance [3–5], which has a profound impact on physical activity and causes falls, which are a major health risk for older adults.

Older adults, who exhibit alterations in muscular activity patterns, muscular atrophy, higher levels of fat infiltration in muscle, appear to have a greater risk of reduced mobility function, which is more pronounced in those persons with LBP [6]. LBP affects an individual’s muscle function especially the core muscles, such as the transverse abdominus (TrA) [7]. It has been reported that the thickness of the TrA was associated with balance stability in individuals with LBP by using the ultrasound imaging [8]. Another study found that the TrA is the first muscle activated and contracted prior to the movement of the limb [9]. Therefore, daily activities such as walking and chair stand tasks, the TrA may play important roles in stabilizing the body and ensuring the successful performance of tasks. And an exercise intervention for strengthening the core muscles (e.g. TrA) seems to be one of the most effective interventions for balance performance in elderly people with LBP.

Different core stability exercise are reported as an effective intervention for LBP. Which mainly including two rehabilitation approaches: (1) a motor control exercises affecting local muscles (lumbar and lateral abdominal muscles) and (2) a general exercise for global body muscles [10]. Recently, motor control exercises affecting local muscles (lumbar and lateral abdominal muscles) have been seen to improve postural stability and/or reduce back problems in young adults [11]. Core stability training has been reported to be an effective intervention for balance performance [12–14]. Szafraniec et al. [12] reported that 4 weeks of core stability training improved balanced performance in health adults. Zou et al. [13] reported that 4 weeks core stability training improved balance ability in young adults with LBP. A meta-analysis conducted by Wang et al. [14] indicated that core stability training can improve the trunk function balance and mobility of the patients with young LBP patients. For elderly people, balance performance was more important in their daily activities. However, there is a lack of evidence regarding the effects of core stability exercises for balance performance in elderly people with LBP.

Previous studies reported that the incidence rate of LBP in females is higher than that in males [15]. LBP has been found to be an important risk factor for reoccurrence of falls in older women [16]. Therefore, the aim of the present study was to investigate the effects of core stability exercises on core muscles and balance function in elderly women with LBP. The proposed hypothesis was that core stability exercises would improve core muscle thickness and mobility function such as timed up-and-go (TUG) test, 10-m walking test (10 M-WT), and four-square step test (FSST) in older women with LBP.

## Materials and methods

### Design

In this triple blinded randomized controlled trial(i.e.the participants,the data analyzer and researchers taking measurements was blinded). 34 elderly people with LBP were recruited as volunteers. This sample was divided into two groups (intervention and control) by random lists. Random grouping was performed using SPSS 20.0 software (fixed value: 20190727). The study protocol was approved by the ethics committee of the First Affiliated Hospital of Sun Yat-sen University in Guangzhou, China. It was done in accordance with the Declaration of Helsinki, good clinical practices, and applicable laws and regulations, and it meets standards of the CONSORT guidelines (approval number#2019469). Written informed consent was obtained from all participants before the experiment.

### Participants

The calculation of statistical power was performed a priori using G Power Software. We estimated the sample size according to the number of two basic averages. According to previous research [17], the balance data of the experimental group were selected as the effect index. With a power analysis set power of 0.80, effect size of 0.75 [18], and 10% loss rate, 12 participants were required for each group.

Participants with LBP were recruited after responding to a recruitment advertisement that was launched through the local community and different activity centers for the elderly. Then they came to the First Affiliated Hospital of Sun Yat-sen University and were diagnosed by a physician specialized in physical medicine and rehabilitation (physiatrist) .

The inclusion criteria were as follows: (1) age ≥ 60 years; (2) nonspecific LBP for at least 3 months in the last year; (3) a Mini-Mental State Examination (MMSE) score > 24 (out of 30) and Montreal Cognitive Assessment (MoCA) score > 26 (out of 30); and (5) Pain score on the visual analog scale (VAS) ≥ 3 (out of 10). Participants with LBP were excluded from the study if any of the following exclusion criteria were met: (1) a history of spinal surgery, rheumatological diseases of the spine, severe spinal pathologies or deformities, or spinal tumors; (2) a history of recent low-extremity injury, endocrine or neuromuscular disease, arthritis or orthopedic disease, orthostatic hypotension, vision problems, vestibular system disease, or any other physical injury that might affect balance; and (3) the use of psychoactive or antihypertensive drugs (antidepressants, antipsychotics, sedatives/hypnotics, antiepileptics, or antiparkinsonian drugs).

### Procedures

**Fig. 1:**
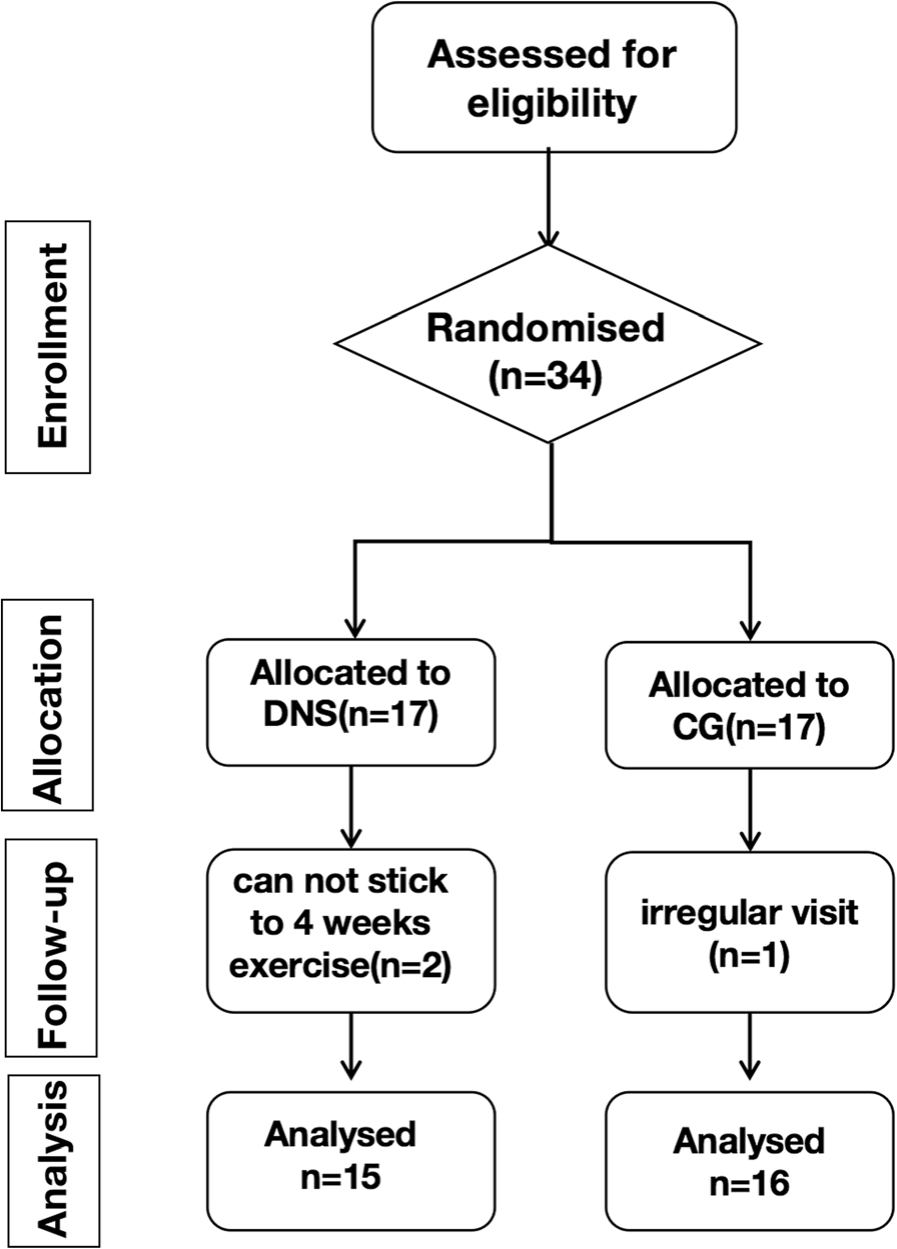
Flowchart showing participate screening and the experimental protocol

The protocol of the present study is showed in Fig. 1.

#### Basic evaluations (sociodemographic evaluation, MMSE, MoCA, VAS, ODI)

The experiment was conducted in bright light in a safe and quiet physiotherapy room. At the beginning, sociodemographic information of each participant was recorded in an individual information sheet. Weight, height, and body mass index (BMI) were also assessed. MMSE and MoCA scale was used to assess the cognitive function. The 10-cm VAS and Oswestry Disability Index (ODI) were used to assess the pain-related clinical outcomes. The whole experiment took approximately 30 min.

##### Pain-related clinical outcomes

Pain intensity was measured by a VAS (0 = no pain, 10 = pain as bad as it could be). The Oswestry disability questionnaire (0 = no disability, 100 = totally disabled) was filled out by the participants. Pain and disability were measured before and after the training [19].

##### ODI

The ODI is commonly used in clinical trials to measure the functional status of participants with LBP [20]. It is comprised of 10 dimensions, with 6 levels being set in each dimension. Specifically, a score of 0 represents the lowest disability level, while 5 indicates the highest disability level. Moreover, the total score is converted into percentage, with a consequent maximum of 100% [21].

##### Congitive function (MoCA and MMSE)

The MMSE and MoCA are both global cognitive screening measures with the same range of scores (0–30), the two tests emphasize different aspects of cognition. The MMSE is a 30-question assessment of cognitive function that evaluates attention and orientation, memory, registration, recall, calculation, language and the ability to draw a complex polygon [22]. The MoCA has six cognitive domains: short-term memory, visuospatial abilities, executive functions, attention and working memory, language, and orientation to time and space. The score range is 0 to 30; the threshold for normal cognitive function is ≧26 [23].

#### Ultrasound imaging measurement

**Fig. 2:**
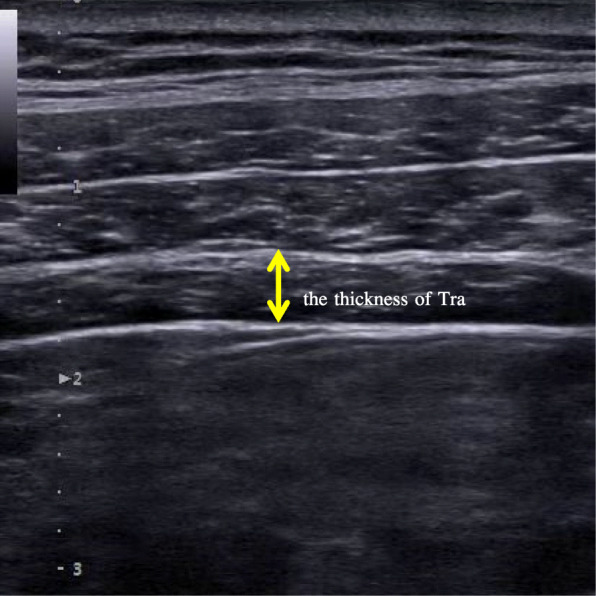
TrA ultrasound image

Ultrasound imaging measurement was conducted before and after the training using SonixGPS ultrasound (SonixTablet, Ultrasonix, Canada) with a 15-MHz linear transducer. To standardize the technique, a supine crook-lying position (hips flexed to approximately 135°, knees flexed to 90°) was adopted by all of the participants [24]. And the evaluations were taken by the same researcher. The transducer was positioned at just above the iliac crest in the midaxillary line. Following a deep inspiration and then a forced expiration, participants were asked to continue to breathe out (further expiration). Images were collected during this period. TrA muscle thickness was measured as the distance between the superior and inferior hyperechoic muscle fascias, at the middle of the ultrasound image (Fig. 2). The ultrasound imaging measurement was repeated three times, and the mean values of were used for data analysis.

#### Mobility functions (TUG, 10 M-WT and FSST)

All the participants completed 3 assessments for mobility functions, which involved the timed up-and-go (TUG) test, 10-m walking test (10 M-WT), and four-square step test (FSST).

The TUG test required the participant to start with standing up from a chair without handles and then walking 3 m as quickly as possible. After that, the participant turned around an obstacle, walked back, and finally sat down on a chair.

In the 10 M-WT test, participants were instructed to walk at their self-perceived comfortable pace. The time to complete 10-m walking was recorded using a stopwatch [25].

**Fig. 3:**
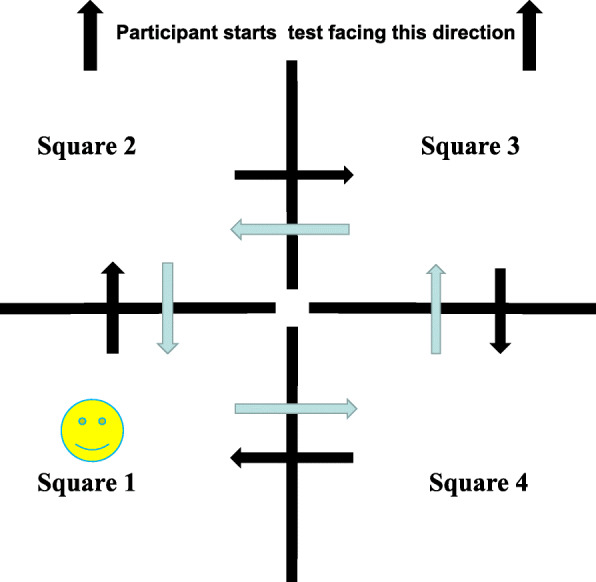
FSST test image

The FSST included four squares, and the participant was required to start in the first square (facing the second square) and step over a cane to enter each additional square while moving in a clockwise direction. They then moved in the counter clockwise direction as quickly as possible without touching the canes (Fig. 3). The trials were repeat if the participant’s foot touched the canes.

In the three dynamic balance tests, participants could have one or two practice trials before each test. Each test was repeated three times and were assigned randomly to the participants. The completion time were recorded during the experiment, and the mean values were used in the following analysis.

### Interventions

All study personnel were trained and certified to implement study protocols in an effort to ensure standardization within and across sites. The intervention period was 4 weeks. Exercise therapists were trained to deliver exercises and physiotherapy.

#### Core stability group

Participants in the training group (TG) received core stability training (25-30 min) + physiotherapy (40 min).

Participants underwent intervention for 4 weeks (4 sessions per week, 65–70 min each session) .

The education session was performed at the clinic by a trained doctor at the first visit. Moreover, a printed pamphlet with instructions on how to perform the exercises was given to each participant.

**Fig. 4:**
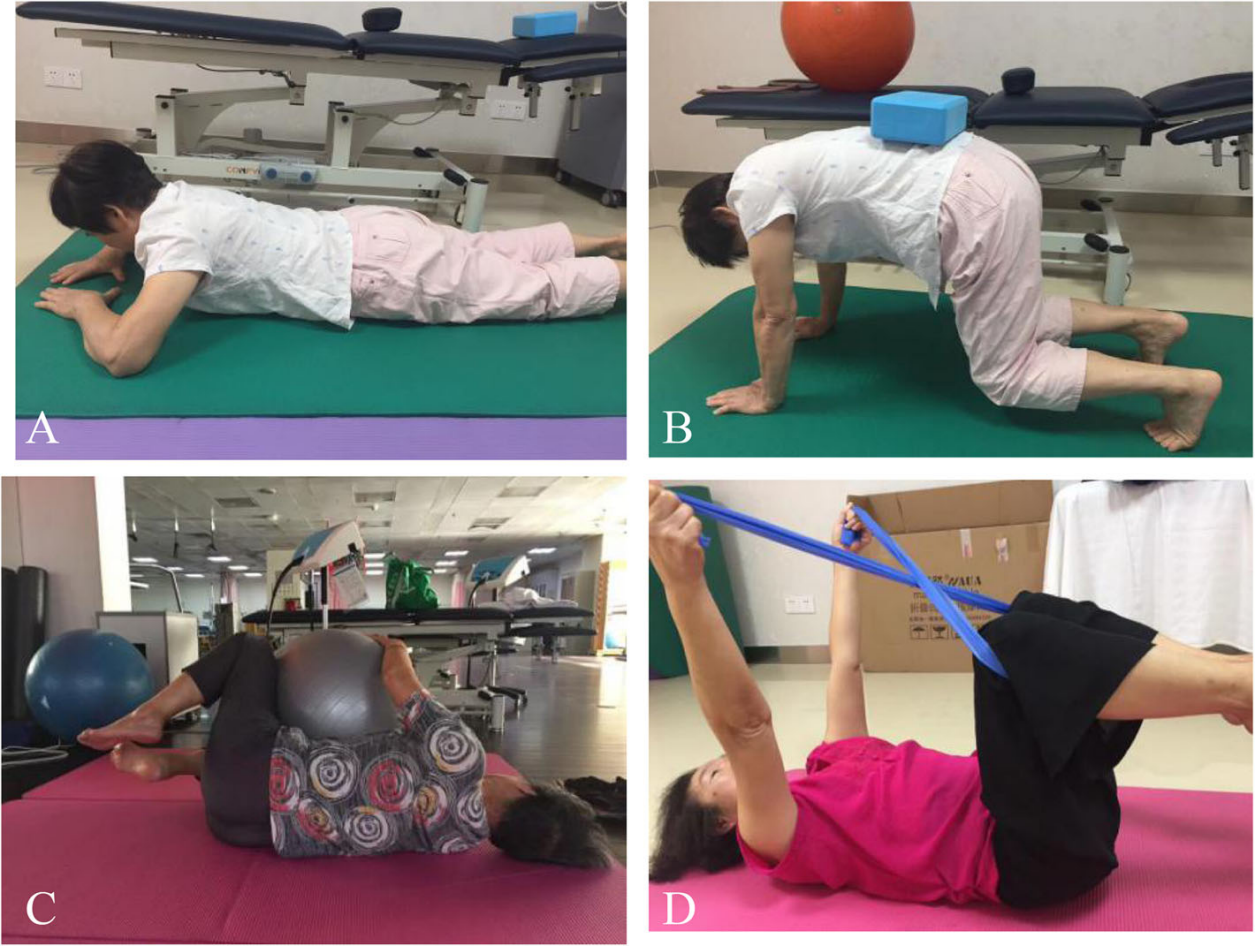
Image of training. Core stabilization exercises: (**A**). crawl position exercise, (**B**). the quadruped exercise with yoga blocks, (**C**). abdomen exercise with swiss ball in supine position (**D**). abdomen exercise with resistance band in supine position

Abdominal respiration skills are learned before the intervention, the exercise movements selected in this study were mainly focus on abdominal muscles such as TrA [14]. Exercises were designed 4 sets, two static exercises (Fig. 4, A and B) and two dynamic exercises (Fig. 4, C and D), from 8 to 10 repetitions and contractions from 10 s to 20 s. Rest intervals were set as 10 s between the sets and 2 mins between the exercises. Core muscle stability training for intervention group included:

1. crawl position exercise (Fig. 4, A)
2. the quadruped exercise with yoga blocks (Fig. 4, B)
3. abdomen exercise with swiss ball in supine position (Fig. 4, C)
4. abdomen exercise with resistance band in supine position (Fig. 4, D)

The physiotherapy treatment included interference wave and magnetic resonance thermal therapy. Two pairs of interference wave electrodes (SK-9SDX, MINATO, Japan) intersected with each other around the painful point of back for 20 min. For magnetic resonance thermal therapy (magneto-vibration heat treatment (LGT-2600B, China), the participants were positioned in a supine position, the foldable electrodes were placed in the participant’s waist pain site, setting mode 1(vibration therapeutic apparatus:50–120 beats/s, temperature:40–58 °C), underwent for 20 min. The training for the TG took about 1 h. There were 4 weeks of training at 4 times per week.

#### CG group

Participants in the CG received physiotherapy that included interference wave + magnetic resonance thermal therapy for a total of 40 min every time at 4 times a week for 4 weeks.

### Statistical analysis

Differences in demographic variables between groups were tested using an independent t-test. Two-way repeated-measures ANOVA was conducted to analyze the data from the ultrasound imaging measurement, VAS, ODI, and dynamic balance tests. The effect size for the statistical analysis was reflected by ƞ2p. The value of low effect size, moderate effect size and high effect size was 0.04, 0.25 and 0.64 [26]. The between-subject factor was the group (TG or CG), and the within-subject factor was time (pre-experiment and post-experiment). Greenhouse-Geisser correction was used when Mauchly’s test of sphericity was violated. Post hoc pairwise comparisons with Bonferroni adjustment were applied when significant interaction effects were observed. The thickness change of Tra was selected to explore the associations between the change of mobility functions. The significance level was set at *P* < 0.05 for all statistical tests. All data were analyzed using the software SPSS 20.0.

## Results

### Participants

Participants’ socio-demographic and health characteristics at baseline are shown in Table 1. Briefly, the sample consisted of 31 adults, with a mean age of 64 years. Of the 34 participants who met the inclusion criteria, 31 participants received a final assessment, and the dropout rate was about 9%. More specifically, 2 participants in the TG were excluded because of they could not commit to 4 weeks exercise, and 1 patient in the CG was excluded because of her irregular visits. Therefore, 15 participants ultimately remained in the TG, and 16 participants remained in the CG for analysis. All the participants recruited in the present study were female. There were no significant differences in age, weight, or height between two groups (*P* ≥ 0.05; Table 1).

**Table 1:** Demographic characteristics of the two groups

Characteristic		TG (*n* = 15)	CG (*n* = 16)	*t-*values	*P-*values
Age (years)		64.60 (3.71)	64.12 (2.96)	0.39	0.69
Sex		Female	Female	–	–
Hand dominance		Right	Right	–	–
Height (m)		1.58 (4.63)	1.57 (3.29)	0.69	0.49
Weight (kg)		58.40 (5.06)	56.06 (3.15)	1.553	0.13
MMSE		28.93 (0.96)	29.06 (0.85)	−0.39	0.69
MoCA		26.66 (0.72)	26.81 (0.83)	−0.51	0.60
Body mass index (kg/m^2^)		23.34 (1.37)	22.72 (1.20)	1.337	0.19
Pain duration (years)		8.40 (3.37)	7.81 (3.05)	0.50	0.61
Academic level	Primary education (0-7 years)	2 (13.33)	0	–	0.28
	Secondary education (8-15 years)	10 (66.67)	11 (73.33)	–	
	Tertiary education (≥16 years)	3 (20)	5 (29.41)	–	

The categorical variables are expressed as n (%), and the continuous variable are expressed as the mean (standard deviations). *kg* kilogram, *m* meter, *MMSE* Mini-Mental State Examination, *MoCA* Montreal Cognitive Assessment, *VAS* visual analog scale, *ODI* Oswestry disability index, *CG* control group, *TG* core stability training group.

### Pre- and post-intervention-related change in all outcome measures

Two-way repeated-measures ANOVA was conducted to analyze the data of TrA thickness, VAS, ODI, and dynamic balance tests. The time effect was not significant in the TrA and FSST parameters [TrA thickness: *F* (1, 29) = 3.36, *P* = 0.077, ƞ2p = 0.104; FSST: *F* (1, 29) = 1.39, *P* = 0.247, ƞ2p = 0.046]. The time effect was significant for VAS, ODI, TUG, and 10 M-WT [VAS: *F* (1, 29) = 26.10, *P* < 0.001, ƞ2p = 0.474, ODI: *F* (1, 29) = 35.24, *P* < 0.001, ƞ2p = 0.549, 10 M-WT: *F* (1, 29) = 6.99, *P* = 0.013, ƞ2p = 0.194, TUG: *F* (1, 29) = 27.14, *P* < 0.001, ƞ2p = 0.483]. The group effect was significant for the VAS, TUG, 10 M-WT, and FSST parameters [VAS: *F* (1, 29) = 12.30, *P* = 0.001, ƞ2p = 0.298, 10 M-WT: *F* (1, 29) = 5.82, *P* = 0.022, ƞ2p = 0.167, FSST: *F* (1, 29) = 5.39, *P* = 0.027, ƞ2p = 0.157, TUG: *F* (1, 29) = 11.65, *P* = 0.002, ƞ2p = 0.287]. The group effect was not significant for TrA and ODI [TrA thickness: *F* (1, 29) = 0.437, *P* = 0.514, ƞ2p = 0.015;ODI: *F* (1, 29) = 0.966, *P =* 0.334, ƞ2p = 0.334]. The time × group interaction effect was significant for TrA thickness [*F* (1, 29) = 5.98, *P* = 0.021, ƞ2p = 0.171], VAS [*F* (1, 29) = 9.05, *P* = 0.005, ƞ2p = 0.238], ODI [*F* (1, 29) = 14.77, *P* = 0.001, ƞ2p = 0.338], FSST [*F* (1, 29) = 9.48, *P* = 0.005, ƞ2p = 0.246], and TUG [*F* (1, 29) = 21.50, *P* < 0.001, ƞ2p = 0.426]. However, the time × group interaction effect was not significant for 10 M-WT: *F* (1, 29) = 3.70, *P* = 0.064, ƞ2p = 0.113.

**Fig. 5:**
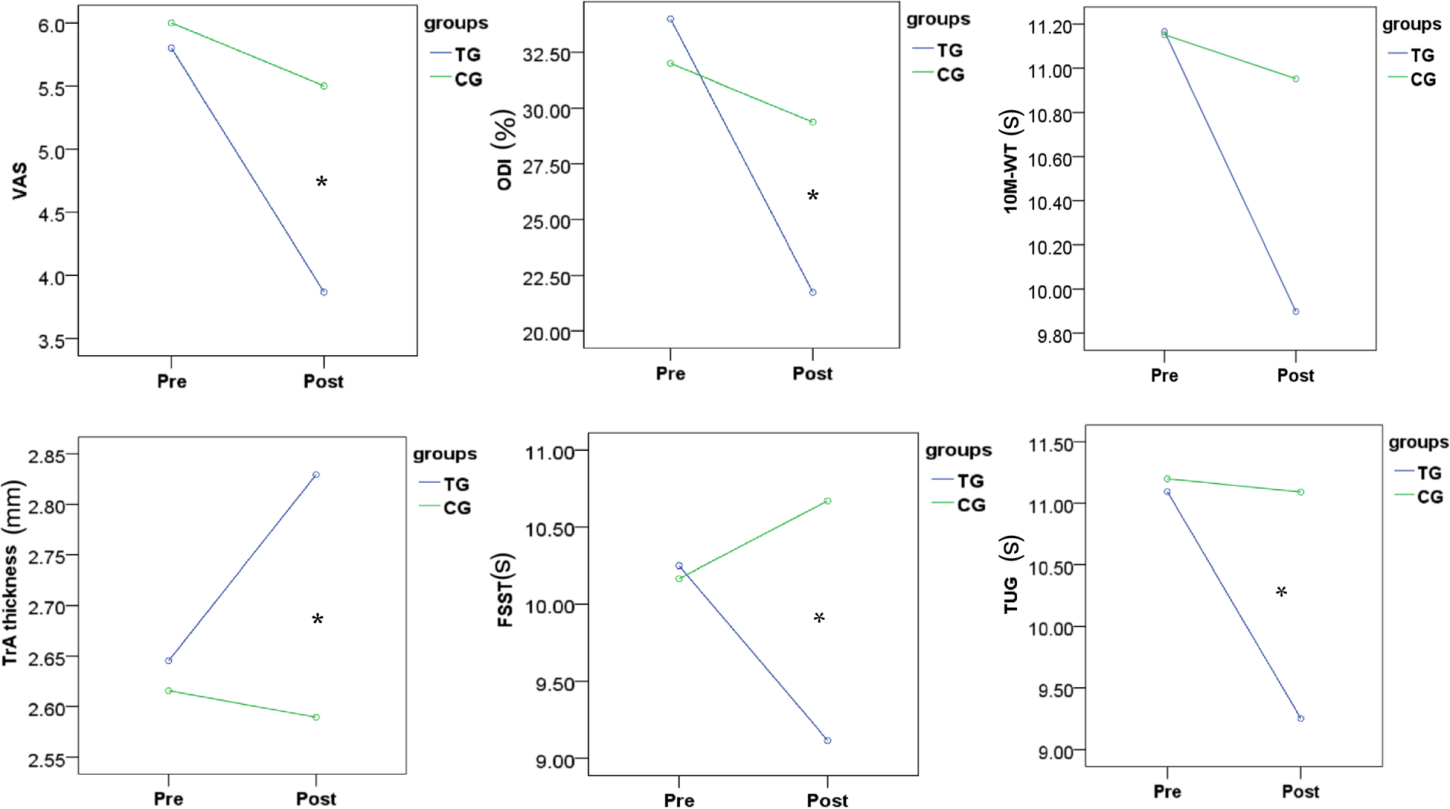
Presents a Pre- and Post-intervention-related change in all outcome measures

Figure5 Pre- and Post-intervention-related change in all outcome measures in TG and CG (* indicate significant interaction effects)

Two-way repeated-measures ANOVA was conducted to analyze the data of TrA thickness, VAS, ODI, and dynamic balance tests (Table 2). As expected, core training and physiotherapy both had positive effects on reducing pain intensity, but there was more reduction in the TG. As for ODI, it only decreased in the TG. Improvements for all the motor functions were observed in only the TG. The TG participants’ TrA thicknesses were significantly changed.

**Table 2:** Outcome variables pre- and post-interventions for two groups

Items	TG		CG	
Outcome variables	Pre	post	Pre	post
TrA	2.64 (0.54)	2.82 (0.51)	2.61 (0.64)	2.58 (0.59)
VAS	5.80 (0.94)	3.80 (1.35)	6.00 (0.89)	5.5 (0.63)
ODI	34.0 (7.78)	21.72 (8.84)	32.00 (9.43)	29.37 (8.66)
TUG	11.09 (0.89)	9.24 (0.90)	11.20 (0.76)	11.09 (1.17)
10 M-WT	11.16 (1.24)	9.89 (0.85)	11.15 (0.69)	10.95 (1.03)
FSST	10.24 (0.86)	9.11 (1.09)	10.16 (1.45)	10.67 (1.08)

*VAS* visual analog scale, *ODI* Oswestry disability index, *CG* control group, *TG* core stability training, *TUG* timed up-and-go, *10 M-WT* 10-m walking test, *FSST* four-square step test, *TrA* transverse abdominal muscle.

### Associations between the change of TrA thickness and the change of dynamic balance scores from pre- and post-interventions for two groups

Associations between the change of TrA thickness and the change of dynamic balance scores from pre- and post-interventions for two groups were shown in Table 3 All the associations between change of TrA thickness and change scores in dynamic balance were not significant (*P* ≥ 0.05).

**Table 3:** Associations between the change of TrA thickness and the change of dynamic balance scores from pre- and post-interventions for two groups

			TUG	10 M-WT	FSST
TrA thickness	Core training group	*Pearson r*	0.165	0.085	0.079
		*P*	0.557	0.764	0.780
	Control group	*Pearson r*	0.091	0.242	0.361
		*P*	0.737	0.366	0.369

## Discussion

The aim of this randomized controlled trial was to estimate the effectiveness of core stability training in older women with LBP. To the best of our knowledge, this is the first clinical trial presenting the effects of a 4-week core stability training in older women with LBP, which makes it difficult to compare our study with any previous study. The results showed that training and physiotherapy can both reduce pain compared to the baseline, but the core stability training was more effective in ODI and TrA thickness than the control treatment. For balance function, the findings of TUG, 10 M-WT, and FSST suggested that TG participants’ dynamic balance performance was significantly enhanced.

TUG, FSST, 10 M-WT tests are reliable and valid fall-risk assessments [25, 27, 28]. The study has found that for healthy elderly people and elderly people with balance dysfunction.the cut-off point time for perform the FSST was 11 s, TUG was 12 s [27, 28]. In this study the TUG and FSST assessment in elderly people with LBP were approached this cut-off point value pre intervention. Improvements for all the motor functions were observed only in the TG indicated that core stability training can be effective in older participants with LBP who may have balance deficits. A meta-analysis conducted by Gamble et al. [29] demonstrated that core stability exercise could improve balance ability in stroke patient. Another study conducted by Saravanakumar et al. [30] found that tai chi and yoga significantly improved the dynamic balance performance in older adults. However Tai chi and yoga are physical activity focus on motor balance and regulate emotion. For elderly people with LBP, who exhibit alterations in muscular atrophy, especially the core muscles such as TrA. The core stability exercise focus on core muscles and motor control seem to be more effective.

In the present study, our sample was older people with LBP. Pain induces spinal motility restrictions, lumbar proprioceptive losses, weakening of lower-extremity sensory feedback, and trunk muscle weakness and atrophy [31–33]. Which could have a profound impact on their motor function. In our study, the pain was significantly decreased in the TG compared to the CG. The previous review showed that exercise-based treatment approaches are most likely to result in improvements in pain and function. Which was consistent with our study.

We assessed TrA thickness to assess the effect of core stability training on older people with LBP. This muscle is one of the important muscles in maintaining the stability and proprioceptive sense of the lumbar trunk. It manages the stability of the body trunk through co-contraction without movement of joints. Our results showed TrA thickness was increased in the core stability training group. Which was consistent with a previous study conducted by Kong et al. [34]. In that study, core exercise training significantly increased the thicknesses of the TrA in LBP young adults. However, Park et al. [35] found no significant change in TrA thickness in LBP young adults after 12 times of core exercise training. A potential reason was that the 12 times of training were insufficient to make significant changes in muscle morphology. For elderly people, who could, benefit more from the training than the untrained young adults [36]. Thus in our study TrA thickness was increased after 16 training sessions four times a week of core exercise training.

Associations between change of TrA thickness and the change of dynamic balance were not significant in our study. These findings were inconsistent with those reported in the previous studies [8, 37, 38]. In Halliday et al’s [37] study, after 8-weeks core exercise training, the change of thickness of the TrA was associated with the change of balance stability. Shamsi et al. [8] reported thicknesses of TrA was associated with balance stability in individuals with LBP. The reasons why we did not find the significant sssociations between change of TrA thickness and the change of dynamic balance in this study might be as follows. First, the potential reasons were that the duration of intervention, in Halliday et al’s [37] study, the duration of the intervention was 8-weeks, whereas in our study, the duration of intervention was only for 4 weeks. The shorter duration might not sufficiently lead to significance association between TrA thickness with balance performance. Second, it might related about use the different tools of balance measurements, in Gong et al’s [38] study, the static balance ability was the outcome meassures. However, in our study we assessed the dynamic balance performance.

Our study addresses several gaps in the knowledge and limitations of previous research in this area. Few studies have focused on core exercise for balance performance in older women with LBP. Given the importance of balance performance for independent living in old age and poor balance being associated with falls risk, the intervention for balance performance in elderly people with LBP is worthy of attention. Our results suggested that 4 weeks of core stability training could enhance the balance ability in elderly people with LBP.

A major limitation of the present study is a lack of follow-ups. Another limitation is that we do not measured the stability of the trunk. Third, this study excluded the participants any other physical injury. The sample recruited in the present study might not be able to represent the general older adults. The future study should consider the effects of any other physical injury on balance. Fourth, the short intervention period may be a limiting factor of this study. In the next study, it will be necessary to examine the effects of core training effect on core muscles by lengthening the study period. Fifth, the study only assess the abdominal muscle, the back muscle such as multifidus were not considered in this study.

## Conclusions

Our study suggests that core stability training is a promising technique to improve associated symptoms and motor balance in older women with LBP.

## Supplementary Information

**Additional file 1.**

## Data Availability

The datasets used and/or analysed during the current study are available from the corresponding author upon reasonable request.

## Abbreviations

LBP: Low back pain
TG: Core stability training group
CG: Control group
TrA: Transverse abdominal muscle
VAS: Visual analog scale
TUG: Timed up-and-go
10 M-WT: 10-m walking test
FSST: Four-square step test
MMSE: Mini-Mental State Examination
MoCA: Montreal Cognitive Assessment
ODI: Oswestry disability index
