# Supplementary material for: Effects of core stability training on older women with low back pain: a randomized controlled trial
Source: Eur Rev Aging Phys Act. 2022 Apr 15;19:10. doi: 10.1186/s11556-022-00289-x (PMC9011968; doi:10.1186/s11556-022-00289-x)
Supplement: Supplementary file 1 — Additional file 1. [file 11556_2022_289_MOESM1_ESM.pdf]

中山大学附属第一医院临床科研和实验动物伦理委员会

IEC for Clinical Research and Animal Trials of the First Affiliated Hospital of Sun Yat-sen University

科研项目伦理审查批件

Approval Letter for Research Protocol

批件号	伦审[2019]469号	审查日期	2019年12月04日
项目名称	动态神经肌肉稳定训练干预老年慢性腰痛平衡功能及跌倒效应的研究		
研究科室	康复医学科	项目负责人	王楚怀
审查类别	■初始审查　□跟踪审查　□复审		
审查方式	会议审查	申办方	N/A
出席情况	出席13人，投票13人，回避0人		
跟踪审查频率	12个月	批件有效期	12个月
审查文件	1. 医学伦理委员会项目评审受理表<br>2. 医学研究项目初始审查申请书<br>3. 知情同意书（1.0，2019年11月20日）<br>4. 研究方案（1.0，2019年11月20日）		
审查意见	1. 经会议审议，该研究符合医学伦理原则及道德要求，研究方案可行，同意按所提交材料开展研究。<br>2. 参与研究的专业科室在项目开展过程中，应遵循已经由本伦理委员会批准的方案执行，应遵守国际《赫尔辛基宣言》及我国的伦理原则、道德标准及相关的法律、法规、常规、制度等。<br>3. 在临床研究过程中，对研究方案和知情同意书等相关文件所做的任何修改，均应提交本伦理委员会再审批，经本伦理委员同意后方可实施。<br>4. SAE/SUSAR报告、方案违背报告须及时递交伦理委员会。本中心发生的SAE必须在24小时以内报告；中国其他中心的SAE，至少每3个月一次及时报告；SUSAR至少每6个月一次报告伦理委员会。方案违背报告应在发现一个月内报告伦理委员会。<br>5. 请按照伦理委员会规定的年度/跟踪审查频率，项目负责人在截止日期前一个月提交年度报告/中期报告；若暂停或提前中止临床研究，请及时提交中止报告。<br>6. 研究完成时，须向本伦理委员会提交结题报告。<br>**本委员会的职责、人员组成和工作程序遵循ICH-GCP、中国GCP，符合赫尔辛基宣言的原则，并遵守中国相关法律法规。**		
主任委员签名	[illegible]		
签发日期	2019年12月6日		
盖章	中山大学附属第一医院临床科研和实验动物伦理委员会		

地址：广州市中山二路58号，中山大学附属第一医院临床科研和实验动物伦理委员会

电话：020-87780263、020-87332200转8221 邮编:510080
